# “One Health” Approach for Health Innovation and Active Aging in Campania (Italy)

**DOI:** 10.3389/fpubh.2021.658959

**Published:** 2021-05-11

**Authors:** Vincenzo De Luca, Giovanni Tramontano, Luigi Riccio, Ugo Trama, Pietro Buono, Mario Losasso, Umberto Marcello Bracale, Giovanni Annuzzi, Rosa Zampetti, Francesco Cacciatore, Giannamaria Vallefuoco, Alberto Lombardi, Anna Marro, Mariarosa Anna Beatrice Melone, Cristina Ponsiglione, Maria Luisa Chiusano, Giancarlo Bracale, Gaetano Cafiero, Aurelio Crudeli, Carmine Vecchione, Maurizio Taglialatela, Donatella Tramontano, Guido Iaccarino, Maria Triassi, Regina Roller-Wirnsberger, Jean Bousquet, Maddalena Illario

**Affiliations:** ^1^Dipartimento di Sanità Pubblica, Università degli Studi di Napoli “Federico II, ” Naples, Italy; ^2^Unità Operativa Semplice Ricerca e Sviluppo, Azienda Ospedaliera Universitaria Federico II, Naples, Italy; ^3^Direzione Generale per la Tutela della Salute e il Coordinamento del Servizio Sanitario Regionale, Naples, Italy; ^4^Dipartimento di Architettura, Università degli Studi di Napoli “Federico II”, Naples, Italy; ^5^Unità Operativa Semplice Microinfusori e tecnologie innovative, Azienda Ospedaliera Universitaria Federico II, Naples, Italy; ^6^Azienda Sanitaria Locale Salerno, Salerno, Italy; ^7^Dipartimento di Scienze Mediche Traslazionali, Università degli Studi di Napoli “Federico II, ” Naples, Italy; ^8^Azienda Sanitaria Locale Napoli 2 Nord, Frattamaggiore, Italy; ^9^Azienda Sanitaria Locale Benevento, Benevento, Italy; ^10^Azienda Sanitaria Locale Avellino, Avellino, Italy; ^11^Dipartimento di Scienze Mediche e Chirurgiche Avanzate e Centro Interuniversitario di Ricerca in Neuroscienze, Università degli Studi della Campania Luigi Vanvitelli, Naples, Italy; ^12^Dipartimento di Ingegneria Industriale, Università degli Studi di Napoli “Federico II”, Naples, Italy; ^13^Dipartimento di Agraria, Università degli Studi di Napoli “Federico II”, Naples, Italy; ^14^Mediterranean Federation for Advancing Vascular Surgery, Naples, Italy; ^15^Unione degli Industriali di Napoli, Naples, Italy; ^16^Federterme, Rome, Italy; ^17^Dipartimento di Medicina, Chirurgia e Odontoiatria, Università degli Studi di Salerno, Salerno, Italy; ^18^Dipartimento di Neuroscienze e Scienze Riproduttive ed Odontostomatologiche, Università degli Studi di Napoli “Federico II,” Naples, Italy; ^19^Dipartimento di Medicina Molecolare e Biotecnologie Mediche, Università degli Studi di Napoli “Federico II,” Naples, Italy; ^20^Dipartimento di Scienze Biomediche Avanzate, Università degli Studi di Napoli “Federico II,” Naples, Italy; ^21^Department of Internal Medicine, Medical University of Graz, Graz, Austria; ^22^MACVIA-France, Fondation Partenariale FMC VIA-LR, Montpellier, France

**Keywords:** health policy, digital health, active and healthy aging, health innovation, future health and health care, information and communication technologies, silver economy

## Abstract

This article describes how innovations are exploited in Campania (Italy) to improve health outcomes, quality of life, and sustainability of social and healthcare services. Campania's strategy for digitalization of health and care and for healthy aging is based on a person-centered, life-course, “One Health” approach, where demographic change is considered capable of stimulating a growth dynamic linked to the opportunities of combining the “Silver Economy” with local assets and the specific health needs of the population. The end-users (citizens, patients, and professionals) contribute to the co-creation of products and services, being involved in the identification of unmet needs and test-bed activity. The Campania Reference Site of the European Innovation Partnership on Active and Healthy Aging is a flexible regional ecosystem to address the challenge of an aging population with a life-course approach. The good practices, developed in the context of research and innovation projects and innovative procurements by local stakeholders and collaborations with international networks, have been allowing the transfer of innovative solutions, knowledge, and skills to the stakeholders of such a multi-sectoral ecosystem for health.

## Introduction

Worldwide demographic change with increasingly aging populations poses a challenge to societies. The Aging Europe report published by the European Commission in 2019 highlighted that the population of older adults (65 years or more) in the European Union (EU) will increase from 101 million in 2018 to 149 million by 2050. The number of people in the EU-28 aged 75–84 years is projected to expand by 60.5%, and those aged 65–74 years to increase by 17.6%, with 9.6% fewer people aged under 55 years living in the EU-28 by 2050 (1). The current COVID-19 pandemic brings an additional challenge for this segment of population, related to the increased risk of adverse outcome in case of infection (2). Furthermore, the deterioration in the quality of life of the older adults as a consequence of loneliness and insufficient treatment resulting from the pandemic imposes health challenges of unknown dimension. Due to the pandemic, health conditions of older adults worsen, and mortality increases, especially among the vulnerable subgroups of populations, setting a country back on its path of human development, and increasing the economic pressure on governments (3). The additional burdens of high unemployment rates among the working-age population and migration will put a strain on health care and welfare systems in southern and eastern European regions (4). The United Nations 2030 Agenda for Sustainable Development (5) calls for a global alliance to address the social challenges that all countries are facing, with a focus on health and training, and in relation to the inequalities that prevent sustainable development for the global society. According to World Health Organization (WHO) Decade for Healthy Aging, if living longer is dominated by poor health, social isolation, or dependency on care, the implications for older people and society are negative in terms of sustainability and development. Poor health for older adults makes them less productive, earn less, retire earlier, and be in greater need for health, care, and social services (6). The impact of social inequalities on population health and mortality has been well**-**documented, as well as the fact that in many western industrialized nations, there is a gradient between social class position (employment, education, and/or income) and the risk of death (7). This has consequences for individuals, industry, public authorities, health and care organizations, policy makers, and investment communities (8). The term “health inequality” refers to the health differences between individuals or groups (9) that are preventable and unnecessary: allowing them to persist is unfair. Differences between social groups are considered health inequalities because they reflect an unfair distribution of health risks and resources. In fact, to be truly effective, health services must be able to manage the health of the entire population and not only those who need it for specific health conditions. The aging process is not the same for all individuals (10): the loss of ability typically associated with aging differs and is developed throughout a person's life-course. Disability and dependency are not an inevitable consequence of aging, and there is a lot we can do to transform it into an opportunity, by supporting and stimulating active and healthy trajectories of aging along the entire life-course. The WHO indicates three key approaches to align health systems to the needs of older populations:

1. Developing and ensuring access to services that are person centered and integrated;
2. Orienting systems around intrinsic capacity; and
3. Ensuring there is a sustainable and trained health workforce.

In this perspective, there is a need to strengthen health promotion and disease prevention along the entire life-course, while addressing the needs of an aging population by supporting independent living at home, self-management of age-related conditions, and reduction of isolation.

## Opportunities From the Internationalization of the Regional Health Systems

The Campania Region follows the indications of the WHO “One Health” approach for the implementation of sustainable development goals across all policies, in an inter-sectoral effort (health, environment, education and training, research, and tourism), including different stakeholders along the quadruple helix of innovation (11, 12). This innovation ecosystem is based on the approach introduced by the European Innovation Partnership on Active and Healthy Aging (EIP on AHA), a stakeholder-driven, voluntary initiative, launched in 2012 to foster innovation and digital transformation in active and healthy aging across countries and regions within the EU (13–15). The EIP on AHA brings together public authorities, health and care providers, researchers, industry, and civic society organizations within, and across, regions to address the challenges of an aging population through the development, implementation, and scaling-up of evidence-based innovative solutions, digital technologies, and models of health and care. One of the biggest challenges for the EIP on AHA is moving innovative solutions to widespread application, beyond projects or test bed environments. The EIP on AHA has been supporting the development and adoption of innovation at scale, either within a region, across a country, or by transferring to other regions and countries. The EIP on AHA Reference Sites (RS) are regional ecosystems that bring together local quadruple helix partners from industry, civil society, academia, and government authorities to focus on a comprehensive, innovation-based approach to active and healthy aging. They are leading regional organizations committed to investing in innovation for active and healthy aging and supporting their transfer and scaling-up across Europe (16). The Reference Site “quadruple helix” structure involves all the stakeholders to work collaboratively and effectively in defining and understanding need; co-creating and co-designing new solutions; offering suitable test bed environments; and evaluating and measuring the impact of innovation on patient, service user, and sustainability. The European Commission (EC) awarded the recognition of “Reference Site” to Campania for the first time in 2012, to the Research and Development Unit of Federico II University Hospital, as the referent for Campania cluster (Table 1). The RS subsequently obtained a first regional recognition (Regional Resolution n. 622/2012), which established a specific coordination group for achieving the objectives of the EIP on AHA. The Federico II University Hospital resubmitted the application to the EC in 2016, obtaining the recognition of “3stars RS.” The Regional Resolution n.221/2017 established an interdisciplinary group for the coordination and management of the RS. The latest “4stars RS” award was obtained in 2019, supported by the Department of Public Health of Federico II University. The scale-up of validated innovative solutions is at the core activity of the stakeholders of the Campania RS of the EIP on AHA (17). Campania RS coalition is also connected to the ProMIS (Programma Mattone Internazionale Salute), the Italian Ministry of Health network for internationalization of regional health systems (RHS). Representatives of all Italian regions participate in ProMIS, to address the current and emerging health needs of the Italian population, sharing and exploiting the opportunities provided by innovative good practices (18).

**Table 1:** The stakeholders of Campania RS along the four helixes of innovation.

**Government/Health providers**	**Research and academia**	**Industry**	**Civil society**
•Campania region, •Local health agency Napoli 1 Centro, •Local health agency Napoli 2 Nord, •Local health agency Napoli 3 Sud, •Local health agency Benevento, •Local health agency Caserta, •Local health agency Avellino, •Local health agency Salerno, •Cardarelli national relevance Hospital, •Pascale national cancer Institute, •Azienda dei Colli national relevance Hospital, •Federico II University Hospital, •Vanvitelli University Hospital, •San Giovanni di Dio e Ruggi d'Aragona University Hospital	•Federico II University of Naples (department of public health, department of advanced biomedical science, department of architecture, department of industrial engineering, department of agricultural science, department of clinical medicine and surgery, department of translational medical science, department of pharmacy, department of molecular medicine and biotechnologies, department of neuroscience, CEINGE advanced biotechnology research center, CIRFF–Interdipartimental research center for pharmacoeconomics and drug utilization), •Parthenope University of Naples (department of physical activities), •University of Salerno (department of medicine and surgery, department of civil engineering), •Suor Orsola Benincasa University of Naples, •Vanvitelli University of Naples (department of medical, surgical and dental specialties) •L'Orientale University of Naples (department of human sciences)	•Digital innovation hub campania (Unione industriali campania), •Federterme (Italian federation of thermal and healing water industries), •Confcooperative Sanità Campania (Confederation of cooperatives–health division)	•Ancel keys institute, •E. Ferrari vocational high school, •Mediterranean federation for advancing vascular surgery, •Italian association Alzheimer disease (AIMA), •Italian cancer association (ANT), •Progetto ALFA association (Health Literacy), •Campus salute (Health promotion), •Federico II University emeritus professors association, •Salute in collina association (general practitioners), •CosmicNet (small and medium municipality network)

The Health Innovation Division of the Campania Region coordinated and supported the stakeholders of the RHS in the development of the ProMIS regional Network (ProMIS@Campania), including public healthcare organizations of Campania, during the 2017–2020 timeframe. ProMIS@Campania and Campania RS collaboratively designed strategic objectives, through:

1. Periodic dedicated meetings, for the recognition of the needs inherent in procedures, tools, and techniques useful for innovation in healthcare (Focus Groups);
2. Involvement in the activities of the national ProMIS network, with particular reference to European initiatives, projects, and working groups; and
3. Thematic workshops.

The Supplementary Materials provide more details on the activities carried out in the framework of the ProMIS@Campania network.

This multidisciplinary, inclusive, and collaborative approach highlighted an asymmetrical progress of the different groups, due to the different levels of maturity of the shared good practices, and to the heterogeneity of the skills expressed by the involved organizations.

The systematic approach described for the strengthening of the ProMIS@Campania regional network has been identified through a decision-making and consensus process integrating evidence gathered from preliminary studies, and an empirical data collection with locoregional stakeholders that was developed iteratively during a 3-year timeframe. This process has allowed the identification of some priority thematic areas for the regional strategies, with respect to which it has been possible to take advantage of the participation in specific European-funded projects, in line with what was developed within the ProMIS@Campania network.

## Campania's Strategic Objectives

The results emerging from the activities of the ProMIS@Campania network indicate, as a strategic objective, the repositioning of the offer of services within a person-centered health ecosystem, through the adoption of innovative approaches that allow the implementation of proactive interventions, and use validated innovative good practices. Campania has important research assets, a key element for the development of an economy based on knowledge, as long as the research results are transferred to the market. “Health is the greatest wealth,” and it is one of the investment sectors with the strongest drive for innovation. Health has a high turnover of knowledge, and it is one of the most important markets for innovation in a public health context. This is particularly true in Campania, where the RHS is leaving from repayment plan and is investing in the enhancement of service provision to citizens, to improve health outcomes and the system sustainability. A proactive, innovation-driven approach translates into opportunities for regional economic growth that can be triggered by digital transformation, enabling cross-sectoral links. The innovation of the Campania ecosystem for health stimulates multisector and multi-actor collaborations (19). The digital transformation strengthens patient empowerment, increasing the accessibility of services for segments of the population with specific health needs, such as the older adults and people with disabilities. The ProMIS@Campania strategy for innovation is aligned with the objectives of the National Health Plan (20) and the new regional Health Plan (21), aimed at capacity building of the regional stakeholders from the social and health systems, strengthening cross-sectoral collaborations at the locoregional, national, and international levels. In this regard, the digital transformation of health and care offers the opportunity to transform social and health challenges, such as the aging of the population and health inequalities into opportunities for sustainable development, and might be one of the priority areas for investments aimed at bridging infrastructural gaps and digital divide (22).

## Emerging Priority Lines of Innovation Needs

The strategic objectives of the ProMIS@Campania network provided a shared vision, facilitating the identification of health needs and supporting the agreement of the local stakeholders on the possible ways to enhance the good practices provided by the collaborative activities within international and national projects. The vision of ProMIS@Campania was aligned with the priorities of the European Commission, allowing the alignment of financial instruments at the regional, national, and European levels for the development of long-term investment plans, to produce structural changes.

**Figure 1:**
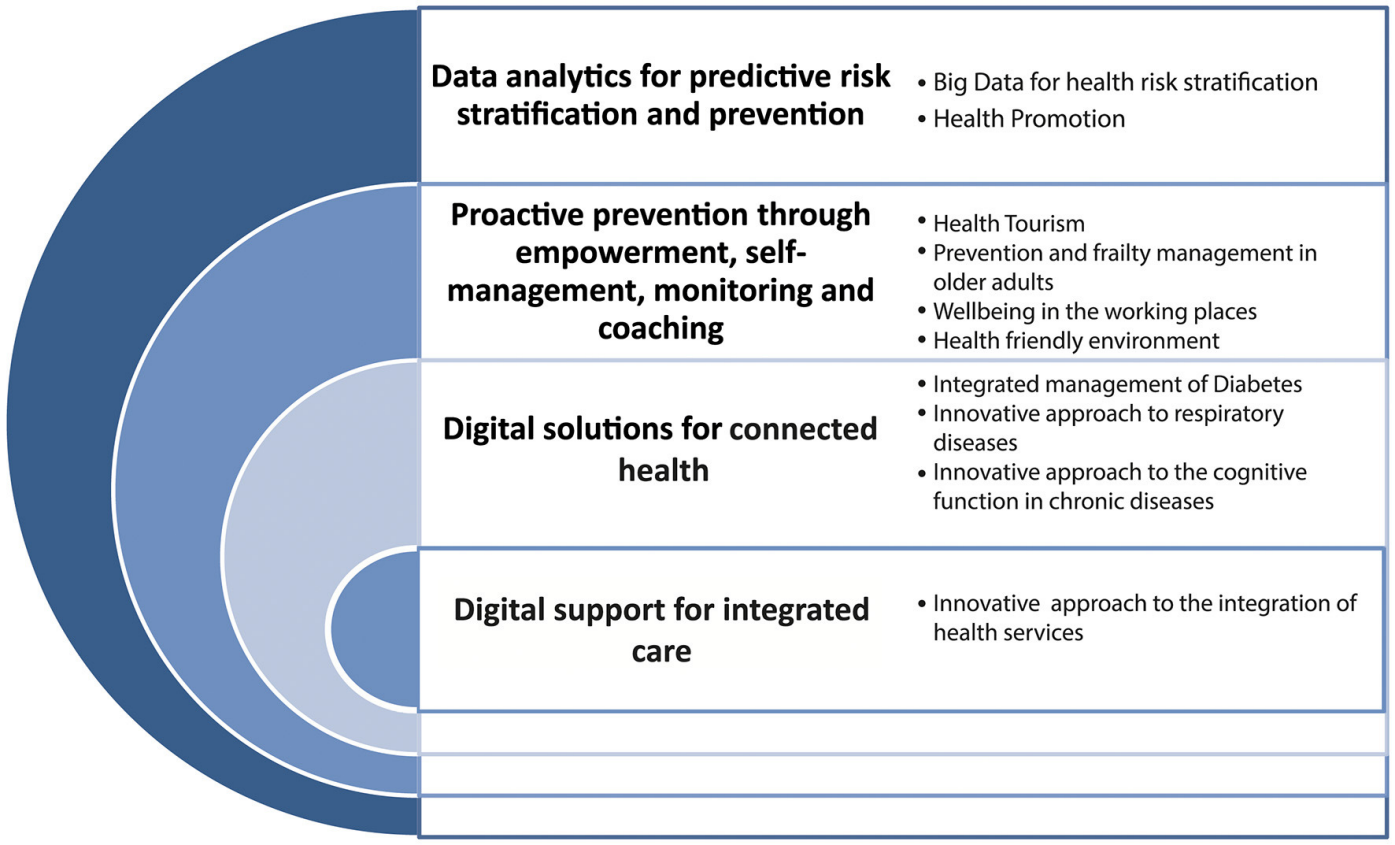
Emerging directories of the health innovation ecosystem in Campania.

Figure 1 represents the directories resulting from the activities carried out within the ProMIS@Campania network, on which the stakeholders of Campania RS have been focusing on, coherently with the four priorities of the European Blueprint for the digital transformation of health and care for the aging society (23, 24).

## The Good Practices for Health Innovation in Campania

Campania participation in the EIP on AHA community broadened the adoption of digital solutions in health and care, exploiting the good practices available (25) through twinning activities with different European regions (Table 2). The twinnings represented an opportunity provided by the EC to exchange knowledge and innovative good practices with high potential for replicability and scaling up (26).

**Table 2:** The good practices exploited by Campania regional health system (RHS) through twinning activities.

**Twinnings**	**Objectives**	**Originator**	**Funding scheme**
QMCI	Early identification of mild cognitive decline	University of Cork- Ireland RS	EIP-AHA scale up strategy- EC
Gastrological approach to malnutrition	Nutritional intervention in individuals with specific health needs	Center for gastrology- Belgium/The Netherlands	EIP-AHA scale up strategy- EC
MASK-ARIA	Prevention and management of allergic rhinitis	MACVIA- France RS	EIP-AHA scale up strategy- EC
Telerevalidatie	Promotion of adapted physical activity in NCCDs patient	Twente RS	EIP-AHA scale up strategy- EC
Listeo+	Management of waiting list and pre-op recommendations	Andalucia region RS	Digital health Europe CSA
My prescription	Polytherapy management and prescription adherence	Andalucia Region RS	Digital health Europe CSA

The participation to international collaborative activities also provided the opportunity to be involved in international research projects to design, test, implement, and monitor the impact of innovative good practices. Such projects were coherent with the priority directories identified by the regional focus groups.

**Figure 2:**
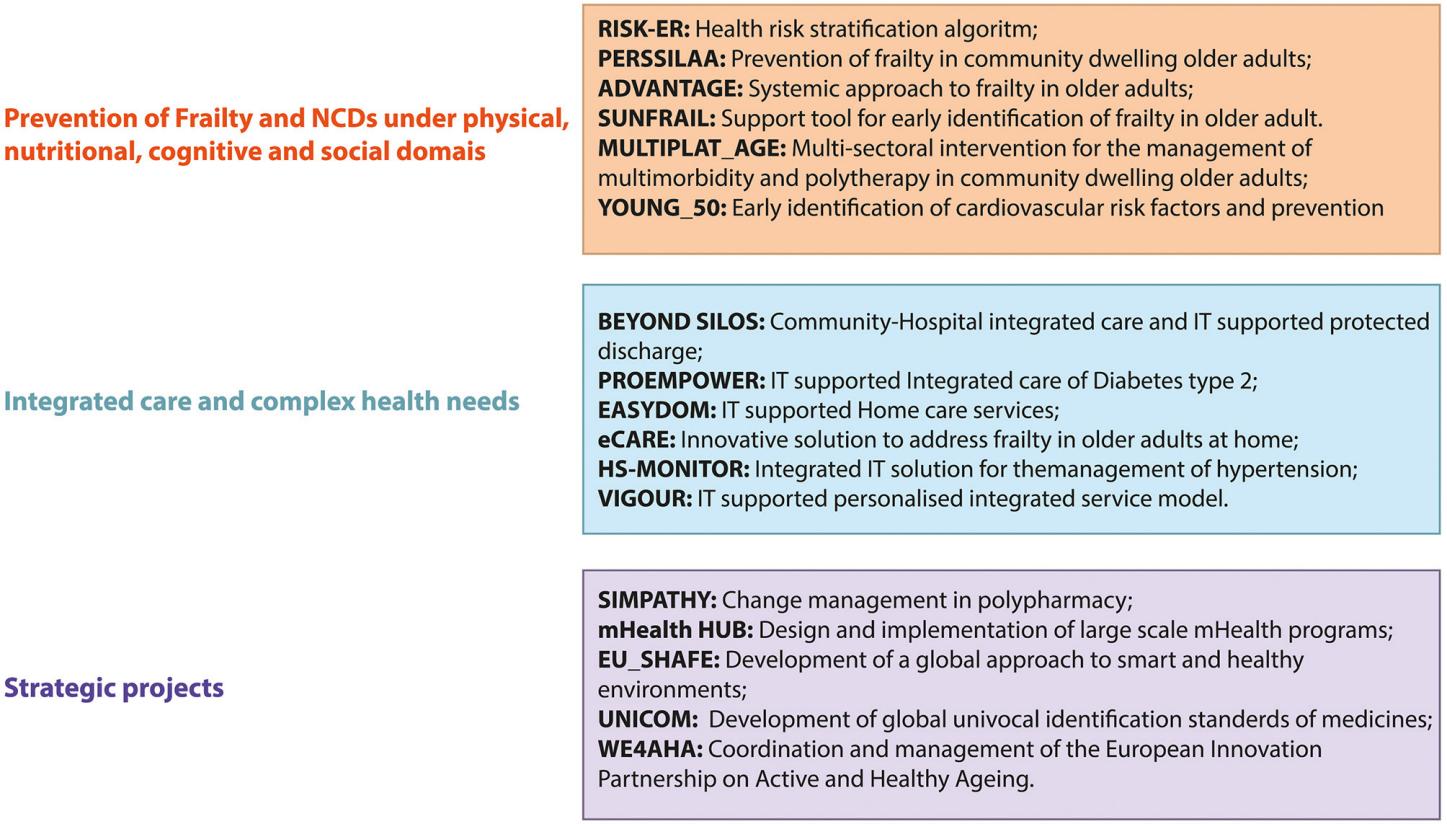
International projects involving Campania's stakeholders.

Figure 2 provides a list of the European projects that were awarded to the stakeholders of Campania RS ecosystem that were subgrouped according to the strategic lines of the ProMIS regional network and have fostered the stakeholders' engagement at different levels in thematic working groups.

## Innovative Procurements

Among the financial instruments for the research and development of digital solutions, a key enabler has been represented by innovative procurements. The EC introduced two forms of public procurement to promote innovation in the public sector: public procurement for innovative solutions (PPI) and pre-commercial procurement (PCP). PPI is a procurement process in which public authorities act as customers for the launch of innovative goods and services that are not yet available on a large-scale commercial basis. PCP is about purchasing research and development (R&D) services rather than ready-to-market products, involving industry players as future suppliers at all stages of solution's development, from research to final product, influencing the market from the demand side, and by stimulating suppliers to develop solutions that respond to well-identified unmet needs of end users (27, 28). The stakeholders of Campania RS community have been taking advantage of the opportunity represented by innovative procurement by joining different consortia, and becoming one of the leading groups in Europe. Innovative procurement supports the improvement of the matchmaking between supply and demand of innovation with the aim of reducing market fragmentation, and promoting a collaborative approach to increase the knowledge sharing and the capacity of the RHS to express its need for innovation in a way that allows interested parties to provide adequate and sustainable solutions. By participating in seven pre-commercial procurements (PCP) (Table 3), the stakeholders of Campania RS have been steering the market toward innovative healthcare solutions and the development of demand-driven products (29). Each PCP offers the opportunity for stakeholders and suppliers to develop specific requirements for innovative solutions, to contribute to clinical pilots in healthcare settings, and to offer vendors the possibility to exploit the results of the pilots for the development of new ready-to-market solutions.

**Table 3:** Pre-commercial procurements in Campania.

**Project**	**Aim**	**Funding (€)**	**Funding scheme**	**Beneficiary**
ProEmpower	Self-management and monitoring of diabetes type 2	750,000.00	European commission	Federico II University Hospital
HSMonitor	Early detection and management of hypertension	924,000.00	European commission	Federico II University Hospital
eCARE	Early detection and management of frailty in older adults	980,000.00	European commission	Benevento local health agency
ADCARE	ICT supported integrated services for multimorbidity management	2,008,125.00	Italian ministry of research	Federico II University Hospital
Ingegneria BioMedica	ICT supported management of medical devices	1,347,500.00	Italian ministry of research	Federico II University Hospital
INCAREHEART	Self-management and monitoring of chronic heart failure	930,000.00	European commission	Federico II University
TIQUE	Integrated approach to chronic heart failure management	1,476,358.33	European commission	Avellino local health agency
	Total	8,415,983.33		

## Innovative Solutions Available in Compania

The innovative solutions tested or under validation concern:

### Stratification of the Risk of Adverse Health Outcomes Through a Validated Algorithm, Based on Regional Data Flows (RISK-ER)

RISK-ER aims to identify the risk of adverse health outcomes in the population through a risk stratification tool and to assess the economic impact associated with negative health outcomes. The Risk-ER tool was developed by the Emilia–Romagna Region, and validated in Campania by the Salerno Local Health Agency on the entire resident population, in collaboration with the University of Salerno. The organizational model for collective and individual interventions is under development (30).

### Integrated IT-Supported Management of Diabetes, for Patient Empowerment and Self-Monitoring, Supporting Adherence to Healthy Lifestyles, Consistent With the Disease (PROEMPOWER)

The solution consists of a shared care plan allowing both patients and professionals to enter data such as glucose measurements, while giving each of them specific rights to do so and integrating data captured directly from devices. Measurement of parameters used by health professionals and patients to manage the disease are transferable to the main ProEmpower solution, including automatic data transfer from device. The solution is, furthermore, able to deliver messages to the patient, including messages formulated by a professional and those automatically generated through data analysis—notifications of deviation from goals, tips for better management, etc. (31).

### Integrated Home Care for Subjects With Multi-Morbidity, Including Low and Medium Intensity of Care (Beyond Silos)

Beyond Silos is an innovative practice that consists of an ICT-based home monitoring system provided as a service by a private home care company, which allows hospital staff to follow up patients at home, as if the patient was still in the hospital (32, 33). The Beyond Silos Good Practice will be recalibrated and used as a basis for the pilot phase of the European VIGOR project.

### Review of the Polypharmacy Regimens (“Medication Review” and “De-prescribing”)

The “FRIENDD” study was implemented to identify cases of prescriptive inappropriateness and report them to the general practitioners connected to a multispecialist team for de-prescribing. The study currently involves experts from different medical areas (pharmacology, geriatrics, neurology, gastrointestinal, and endocrinology) (34).

### A Multisectoral Path for Rhinitis and Its Multimorbidity

The MASK-ARIA initiative aims to develop an interprofessional care model (pharmacists, general practitioners, and specialists) for an integrated management of patients with allergic rhinitis. MASK-ARIA in Campania is developed through a smartphone app that allows for rhinitis assessment and uses a clinical decision support system (CDSS) (35–38).

### The Gastrological Approach to Malnutrition

The gastrological approach to malnutrition is a primary prevention approach to malnutrition in cases of specific nutritional needs, in all healthcare settings (39). The focus of the gastrological approach is the implementation of personalized ICT-supported nutritional interventions that leverage validated screening, assessment, and monitoring tools, recognizing a coherent set of activities aimed at improving food intake in frail individuals.

### Early Identification of Mild Cognitive Decline to Enable Targeted Interventions

A multicenter study was carried out in Campania to validate the Italian version of the Quick Mild Cognitive Impairment (QMCI-I) tool and to obtain normative data. Further studies on Italian patients with MCI (mild cognitive impairment) are underway to investigate the diagnostic properties of QMCI-I, and to use the multidomain structure of the tool to classify the various subtypes of MCI and indicate interventions, including memory training supported by digital tools (40, 41).

### PERsonalized ICT Supported Service for Independent Living and Active Aging

PERSSILAA services are offered to older adults through living labs organized in local communities, and are integrated with healthcare services. This new multimodal service model, focused on nutritional, physical, cognitive, and social dimensions, is supported by an interoperable ICT service infrastructure and by a level of gamification. An extension of the pilot, aimed at Campania rural areas, is currently in the planning and implementation phase (42, 43).

### ICT Supported Adapted Physical Activity

The Telerevalidatie.nl® platform supports the physical activity of sedentary patients at their premises. It provides ICT-supported personalized training program with tutorial videos and at the same time allows to track their use of the platform (44). The Federico II University Hospital has activated a clinic for the physical activity on prescription (PAP), to ensure that patients discharged by a rehabilitation pathway are enrolled in a personalized physical activity program. To this aim, patients who are enrolling in the program are subjected to physical need assessments, performed according to the guidelines for exercise testing and prescription of the American College of Sports Medicine (45). Telerevalidatie.nl® is an example of successful transfer of an innovation to current practice. The solution was initially developed in the framework of the PERSILAA project. Its transfer was supported by two international twinnings that support knowledge transfer and adaptations that resulted in the final integration of the program for adapted physical activity into service provision of the Federico II University Hospital.

### Early Identification of Frailty in Primary and Secondary Care Settings

The SUNFRAIL tool enables early identification of frailty-risk domains in the elderly, in order to prevent functional decline and adverse health outcomes. The tool is currently being used in pilots at the thematic focus group of the ProMIS@Campania network (46).

## Transferable Elements

### New Skills for New Jobs

Tackling the challenge of an aging population requires moving from a reactive approach to disease to a proactive one for health and well-being. The Campania RS approach contributed to the connect multidisciplinary skills and knowledge, placing the citizen at the center of healthcare services, improving their quality and accessibility. As COVID-19 emergency demonstrated, digital literacy and training will be essential among professionals across sectors, for e.g., for secondary use of data, hence, the need to make digital tools and solutions accessible to these groups (47), fostering technological skills. Further strengthening of human resources in a regional health system is pivotal to develop capacity building, integrating the enrollment of new professionals in a continuous training courses, as a fundamental tool for maintaining high-quality standards in the provision of health and social services and protecting individual and collective health.

### Innovation to Market in Campania

The Campania RS ecosystem has been making efforts to implement the EIP on AHA Innovation to Market (I2M) strategy by establishing a dialogue with industrial stakeholders, such as the Digital Innovation Hub of Campania (DIH) and the HealthTech Innovation Hub. Campania DIH is the regional network of private companies engaged in innovation and digital transformation projects, not only in the health sector. The HealthTech Innovation Hub is the result of the collaboration between the Federico II University of Naples and Medtronic Italy, with the aim of combining academic and industry skills to generate new solutions in healthcare. The shared objective is too contrite to the development of the “Digital Health” market in response to the health needs of citizens. This collaborative and cross-sectorial approach follows the implementation of the European plan for “Innovation to market” (I2M) (48) in Campania and broadens the commitment of local stakeholders in the activities and further progress of the I2M plan. The RS can contribute to the organization of activities aimed at enriching and extending the impact of the I2M plan at the regional level. The alignment between the objectives of interested stakeholders strengthens the strategy of the digital market and extends the priorities of digital development in Campania to the health sector. Through this synergy, Campania RS will stimulate and promote the demand for innovation in the healthcare system, improving the level of multidisciplinary knowledge and increasing the awareness of the opportunities offered by digitalization to industry and promoting the adaptation and large-scale adoption of validated good practices.

### Health, Accessible, and Sustainable Tourism

Health is an important sector of the economy, and health tourism is growing rapidly across Europe. Health tourism refers to any form of tourism development related to health creation that includes health promotion, specific health services, or services addressing health needs related to specific conditions (49). An effective approach needs integration between health, social, and tourism services to make tourist destinations accessible, addressing the different pattern of needs: frailty, dependency, health-specific needs that determine spending priorities of significant parts of the general consumer economy, such as the “Silver Economy” (47). The Collaboration between Italian and European Reference Sites, in the context of the national ProMIS network and the EIP on AHA Reference Sites Collaborative Network (RSCN) (50) led to synergic actions in the field of health, to support the development of accessible and sustainable tourism. This is through sharing validated and innovative experiences that strengthen accessibility to health services in the regional, national, and international contexts. Broad, collaborative activities will allow the identification of shared strategies and priority implementation areas to further drive the development of this emerging sector.

### Bottom–Up Approach

Often, the regions adopt a top–down strategy to the development of the local network for innovation, with a centralized definition of priorities, and subsequent collection of good practices on the ground. ProMIS carried out an assessment focusing on the methodology for the development of the local network for the implementation of regional policies in AHA (51). In this study, for example, the strategy of Friuli Venezia Giulia RS is based on the implementation of a regional program for the promotion of healthy aging, with many areas of interventions, such as civil commitment, culture, and social tourism, the access to new technologies, information, and services. A specific panel has been created to implement and manage all those areas of intervention. Liguria RS created a public–private partnership for active aging that collects some of the excellences active in the region in terms of research and innovation (University), social sector, IT companies, training institutions, regional health agencies, and local institutions. Lombardy RS is based on the connection between the regional Directorate for Welfare and the Lombardy Cluster for Technologies in life environments that is a multidisciplinary technological cluster, funded by the Directorate for Productive Activities and Research. In the framework of “Silver Constellation for Health,” Piedmont RS developed an operational setting of interlaced good practices on AHA, important experiences contributing to the health policy, adding new professional and community skills. The Emilia–Romagna RS is composed of a wider regional coordination team involving the Department of Health and Social Policies, the Department of Production Trade and Tourism and ASTER, the technological cluster of the Emilia–Romagna Region.

Campania is following Kotter's eight-step change management model to realize the vision of the “One Health” approach (52, 53). The innovation ecosystem, through Campania EIP on AHA RS and ProMIS@Campania network, engaged a group of stakeholders in the identification and uptake of good practices in the region and beyond (Step 1–2). Sharing knowledge and opportunities of collaboration among stakeholders allowed the development of a shared vision and strategy that were appropriately communicated through newsletters, conference presentations, and scientific publications. This approach empowered others to act on the vision (Step 3–4). The involvement of stakeholders in the regional, national, and European level activities empowered and stimulated other stakeholders (Step 5). The generated good practices represent short-term wins (Step 6), for which it is necessary to consolidate the results and produce more changes (Step 7). The wider implementation and the continuous monitoring of the results will connect the solutions together in a single process of change and anchor innovative approaches to the local culture and context (Step 8).

## Bottlenecks

Innovation of health and care poses many challenges that cannot be addressed timely by an isolated approach, i.e., finance and insurance, technological standards, degree of interoperability of solutions, issues around data security, and solutions for data analytics/data mining, lack of ICT literacy, lack of continuity between different research maturity level (e.g., TLR 1 to 9).

### Limited International Collaborations

The quadruple helix of innovation for the digital transformation of health and care includes also research, and indeed, in Campania, an effort was carried out to strengthen interdisciplinary approaches in expert groups, in all projects and twinnings, but further developments are necessary to ensure that horizontal activities succeed in facilitating the mainstreaming along international opportunities, for example, connecting with international networks. This element is pivotal to reduce fragmentation in the procurement of innovative solutions for health services. Strengthening collaborations at international levels is a key enabler to foster national alliances, facilitate peer-to-peer learning and transfer of innovative, validated good practices. Within this approach, networks play a catalytic role to facilitate capacity building.

### Absence of Digital Services in Standard Care Pathways

Ensuring appropriateness is pivotal to use digital solutions in healthcare services not at a discretion, as they are included in the standard care pathways (Percorsi Diagnostico-Terapeutico e Assistenziali–PDTA), adapted to the specific needs of the person (Piano Assistenziale Individuale–PAI), hence, the efforts of the national health system to establish a nomenclature of digital services and define new PDTA (health and social) for the integrated management of diseases in the territory, when they are available as IT-supported services, hence, the introduction of essential levels of digital healthcare in the new Minimum Service Level (Livelli Essenziali di Assistenza–LEA), with reimbursement arrangements specific for digital services, is pivotal (54).

### Low Digital Literacy

Large-scale adoption of a digital solution for health and care needs to reach high levels of digital literacy for end users (professionals, patients, and citizens) and to consider how digital tools and solutions can be made completely accessible to these groups (47). Only by starting from the analysis of the needs expressed by citizens and institutions will we be able to enhance the market of innovative solutions, responding to concrete unmet needs of older adults and removing the organizational, technological, and behavioral obstacles that influence large-scale adoption in the locoregional context.

### Fragmented Approach to Structural Reform

Health innovation needs strong institutions capable of implementing health reform, with accompanying clear programming objectives and successful projects. Many of the gaps identified are linked to structural reform issues that are difficult to address through project-based funding. Stronger capacity and technical expertise to implement and design reforms can complement the capacity to develop high-quality policies and projects (55).

## Conclusions

The governance approach of Campania RS to address the needs of an aging population translates in strengthening disease prevention and health promotion in the entire life-course through empowerment and digital solutions, and in supporting independent living at home and in community, strengthening self-management of age-related conditions and reducing isolation. Such proactive, user-centered system empowers citizens and informal caregivers to take greater responsibility for health and well-being, by providing information and advising on healthy lifestyles, to stay active and healthy as long as possible (56–58). Similarly, age-friendly environments are beneficial to individuals and society throughout the entire life-course (59) and are enabled by digital solutions. A “Silver Economy,” deployed in the framework of a life-course approach, could stimulate sustainable growth in Campania, creating opportunities and jobs, through digital innovation and across sectors such as transportation, constructions, and built environment, as well as supporting patients and older adults to remain independent, active, and live longer in their homes and communities, while increasing the efficiency and effectiveness of health and social care systems (47). End users are at the center of such approach, to accelerate the design of user-friendly products and services, sharing experiences with new generations, and being involved in test-bed activities (60).

## Author Contributions

MI conceived the presented idea and drafted the table of contents. MI, VDL, and GT wrote the entire manuscript. LR, UT, and PB contributed to the Abstract and the section Strategic Objective. ML, MLC, GB, DT, and AC contributed to the Abstract and the section Health, Accessible, and Sustainable Tourism. GA, UMB, RZ, FC, and MTa contributed to the sections The Good Practices for Health Innovation in Campania, Emerging Priority Lines of Innovation Needs, and Innovative Solutions Available in Campania. GI, AL, and AM contributed to the sections Innovative Procurements and The opportunity of Aging in Campania: new skills, new jobs. GV and MM contributed to section Opportunities From the Internationalization of the RHS. CP and MTr contributed to the section Long-Term Sustainability of Investments in Digital Transformation. CV and GC contributed to the section Innovation to Market in Campania. RR-W and JB contributed to sections Introduction and Conclusions and critical reading. All authors contributed to the article and approved the submitted version.

## Conflict of Interest

The authors declare that the research was conducted in the absence of any commercial or financial relationships that could be construed as a potential conflict of interest.

## References

[1] Strandell H Wolf P. Ageing Europe—Looking at the Lives of Older People in the EU. Luxembourg: Publications Office of the European Union (2019).

[2] Iaccarino G Grassi G Borghi C Ferri C Salvetti M Volpe M. Age and multimorbidity predict death among COVID-19 patients: results of the SARS-RAS study of the Italian society of hypertension. Hypertension. (2020) 76:366–72. 10.1161/HYPERTENSIONAHA.120.15324 32564693

[3] Marois G Muttarak R Scherbov S. Assessing the potential impact of COVID-19 on life expectancy. PLoS ONE. (2020) 15:e0238678. 10.1371/journal.pone.0238678 32941467 PMC7498023

[4] England K Azzopardi-Muscat N. Demographic trends and public health in Europe. Eur J Public Health. (2017) 27 (suppl_4):9–13. 10.1093/eurpub/ckx159 29028238

[5] United Nations. Resolution n. A/70/L.1 Adopted by the General Assembly on 25 September 2015. Transforming Our World: the 2030 Agenda for Sustainable Development. United Nations (2015). Available online at: https://www.un.org/en/development/desa/population/migration/generalassembly/docs/globalcompact/A_RES_70_1_E.pdf (accessed January 22, 2021).

[6] WHO Executive Board. Recommendation n. EB146/23. Report on the Proposal for a Decade of Healthy Ageing 2020–2030. WHO Executive Board (2019). Available online at: https://apps.who.int/gb/ebwha/pdf_files/EB146/B146_23-en.pdf (accessed January 22, 2021).

[7] Arcaya MC Arcaya AL Subramanian SV. Inequalities in health: definitions, concepts, and theories. Glob Health Action. (2015) 24:27106. 10.3402/gha.v8.27106 26112142 PMC4481045

[8] Directorate-General for Research and Innovation European Commission. Population Ageing in Europe: Facts, Implications and Policies. Luxembourg: Publications Office of the European Union (2014).

[9] Kawachi I Subramanian SV Almeida-Filho N. A glossary for health inequalities. J Epidemiol Community Health. (2002) 56:647–52. 10.1136/jech.56.9.647 12177079 PMC1732240

[10] WHO. Integrated Care for Older People: Guidelines on Community-Level Interventions to Manage Declines in Intrinsic Capacity. Geneva: World Health Organization (2017). 29608259

[11] Acharya S Lin V Dhingra N. The role of health in achieving the sustainable development goals. Bull World Health Organ. (2018) 96:591–591A. 10.2471/BLT.18.221432 30262936 PMC6154063

[12] Borrmann M Lindner S Hofer-Fischanger K Rehb R Pechstädt K Wiedenhofer R. Strategy for deployment of integrated healthy aging regions based upon an evidence-based regional ecosystem—the styria model. Front Med. (2020) 7:510475. 10.3389/fmed.2020.510475 33117826 PMC7550727

[13] European Commission. Comunicacation n. COM 2010 546: Europe 2020 Flagship Initiative: Innovation Union. European Commission (2010). Available online at: https://ec.europa.eu/research/innovation-union/pdf/innovation-union-communication_en.pdf (accessed February 23, 2021).

[14] Lagiewka K. European innovation partnership on active and healthy ageing: triggers of setting the headline Target of 2 additional healthy life years at birth at EU average by 2020. Arch Public Health. (2012) 70:23 10.1186/0778-7367-70-23 23088612 PMC3492155

[15] European Commission. Communication n. COM 2012 83: Taking forward the Strategic Implementation Plan of the European Innovation Partnership on Active and Healthy Ageing. European Commission (2012). Available online at: https://eur-lex.europa.eu/legal-content/EN/TXT/PDF/?uri=CELEX:52012DC0083&from=EN (accessed February 23, 2021).

[16] European Commission. European Innovation Partnership on Active and Healthy Ageing – Reference Sites. European Commission (2021). Available online at: https://ec.europa.eu/eip/ageing/reference-sites_en (accessed February 23, 2021).

[17] Bousquet J Michel J Strandberg T Crooks G Iakovidis I Iglesia M. The European innovation partnership on active and healthy aging: the European geriatric medicine introduces the EIP on AHA column. Euro Geriatr Med. (2014) 5:361–434. 10.1016/j.eurger.2014.09.010

[18] Illario M De Luca V Tramontano G Menditto E Iaccarino G Bertorello L. The Italian reference sites of the European innovation partnership on active and healthy ageing: progetto mattone internazionale as an enabling factor. Ann Ist Super Sanita. (2017) 53:60–9. 10.4415/ANN_17_01_12 28361807

[19] Herzlinger RE. Why innovation in health care is so hard. Harv Bus Rev. (2006) 84:58–66:156. 16649698

[20] Conferenza Stato-Regioni. Intesa, Ai Sensi Dell'articolo 8, Comma 6, Della Legge 5 Giugno 2003, n. 131, Tra Il Governo, Le Regioni e le Province Autonome di Trento e di Bolzano Concernente il Patto Per la Salute Per Gli Anni 2019–2021. Rome: Conferenza Stato-Regioni (2019)

[21] Regione Campania. Decreto del Commissario ad Acta n. 94/2019. Piano Triennale 2019-2021 di Sviluppo e Riqualificazione Del Servizio Sanitario Campano Ex Art. 2, Comma 88, Della Legge 23 Dicembre 2009, n. 191. Naples: Regione Campania (2019)

[22] Roberts JP Fisher TR Trowbridge MJ Bent C. A design thinking framework for healthcare management and innovation. Healthcare. (2016) 4:11–4. 10.1016/j.hjdsi.2015.12.002 27001093

[23] WE4AHA Project Consortium. Blueprint on Digital Transformation of Health and Care for the Aging Society. WE4AHA Project Consortium (2018). Available online at: https://ec.europa.eu/digital-single-market/en/blueprint-digital-transformation-health-and-care-ageing-society (accessed January 22, 2021).

[24] Patalano R De Luca V Vogt J Birov S Giovannelli L Carruba G. An innovative approach to designing digital health solutions addressing the unmet needs of obese patients in Europe. Int J Environ Res Public Health. (2021) 18:579. 10.3390/ijerph18020579 33445561 PMC7826628

[25] Yock P Zenios S Makower J Brinton T Kumar U Watkins F. Biodesign: The Process of Innovating Medical Technologies. 2nd ed. Cambridge: Cambridge University Press (2015). 10.1017/CBO9781316095843

[26] Birov S Lavin C Stroetmann V. Scaling-up of ICT solutions in active and healthy ageing through twinning actions. In: Proceedings of the 5th International Conference on Information and Communication Technologies for Ageing Well and e-Health (ICT4AWE), Vol. 1. Heraklion (2019). 10.5220/0007722702220229

[27] European Commission. Decision C 2019 4575 2019: Horizon 2020 Work Programme 2018–2020. European Commission (2019). Available online at: https://ec.europa.eu/transparency/regdoc/rep/3/2019/EN/C-2019-4575-F1-EN-MAIN-PART-1.PDF (accessed January 22, 2021).

[28] European Commission. Communication n. COM 2007 799: Pre-commercial Procurement: Driving Innovation to Ensure Sustainable High Quality Public Services in Europe. European Commission (2007). Available online at: https://eur-lex.europa.eu/LexUriServ/LexUriServ.do?uri=COM:2007:0799:FIN:EN:PDF (accessed January 22, 2021).

[29] De Luca V Birov S Beyhan O Robinson S Sanchez-Nanclares G Acuña M. European specifications for value-based pre-commercial procurement of innovative ICT for empowerment and self-management of diabetes mellitus patients. In: Proceedings of the 5th International Conference on Information and Communication Technologies for Ageing Well and e-Health (ICT4AWE), Vol. 1. Heraklion (2019). 10.5220/0007638700190027

[30] Louis DZ Robeson M McAna J Maio V Keith SW Liu M. Predicting risk of hospitalisation or death: a retrospective population-based analysis. BMJ Open. (2014) 4:e005223. 10.1136/bmjopen-2014-005223 25231488 PMC4166245

[31] De Luca V Birov S Beyhan O Robinson S Sanchez-Nanclares G Acuña M. Developing a digital environment for the management of chronic conditions: the proempower experience of a horizon 2020 PCP for type 2 diabetes. In: Ziefle M, Maciaszek L, editors. Information and Communication Technologies for Ageing Well and e-Health, ICT4AWE 2019, Vol. 1219. Cham: Springer (2020). 10.1007/978-3-030-52677-1_1

[32] Beyond Silos Consortium. Beyond Silos: Taking Integrated Care One Step Further. Beyond Silos Consortium (2016). Available online at: https://beyondsilos.eu/norm/home (accessed January 22, 2021).

[33] Visco V Finelli R Pascale AV Mazzeo P Ragosa N Trimarco V. Difficult-to-control hypertension: identification of clinical predictors and use of ICT-based integrated care to facilitate blood pressure control. J Hum Hypertens. (2018) 32:467–76. 10.1038/s41371-018-0063-0 29713051 PMC6057905

[34] Cataldi M Giallauria F Simeone G Arcopinto M de Luca V del Giudice C. Evidence-based directions for polypharmacy revision: the experience gained by the Campania reference site in the framework of the eip-aha action group A1 and of the friendd pilot study. In: Costa E, Giardini A, Monaco A, editors. Adherence to Medical Plans for Active and Healthy Ageing. Nova Science Publisher, Inc. (2017). p. 41–56.

[35] Bousquet J Bedbrook A Czarlewski W Onorato GL Arnavielhe S Laune D. Guidance to 2018 good practice: ARIA digitally-enabled, integrated, person-centred care for rhinitis and asthma. Clin Transl Allergy. (2019) 9:52. 10.1186/s13601-019-0291-6 30911372 PMC6413444

[36] Bousquet J Anto JM Bachert C Haahtela T Zuberbier T Czarlewski W. ARIA digital anamorphosis: digital transformation of health and care in airway diseases from research to practice. Allergy. (2021) 76:168–190. 10.1111/all.14422 32512619

[37] Bousquet J Anto JM Haahtela T Jousilahti P Erhola M Basagaña X. Digital transformation of health and care to sustain planetary health: the MASK proof-of-concept for airway diseases-POLLAR symposium under the auspices of Finland's presidency of the EU, 2019 and MACVIA-France, global alliance against chronic respiratory diseases (GARD, WH0) demonstration project, reference site collaborative network of the european innovation partnership on active and healthy ageing. Clin Transl Allergy. (2020) 10:24. 10.1186/s13601-020-00321-2 32577216 PMC7304084

[38] Bousquet J Schünemann HJ Togias A Bachert C Erhola M Hellings PW. Next-generation allergic rhinitis and its impact on asthma (ARIA) guidelines for allergic rhinitis based on grading of recommendations assessment, development and evaluation (GRADE) and real-world evidence. J Allergy Clin Immunol. (2020) 145:70–80.e3. 10.1016/j.jaci.2019.06.049 31627910

[39] Illario M Maione A Rusciano M Goessens E Rauter A Braz N. NutriLive: an integrated nutritional approach as a sustainable tool to prevent malnutrition in older people and promote active and healthy ageing—the EIP-AHA nutrition action group. Adv Public Health. (2016) 2016:5678782. 10.1155/2016/5678782

[40] Iavarone A Carpinelli Mazzi M Russo G D'Anna F Peluso S Mazzeo P. The Italian version of the quick mild cognitive impairment (Qmci-I) screen: normative study on 307 healthy subjects. Aging Clin Exp Res. (2019) 31:353–60. 10.1007/s40520-018-0981-2 29949025

[41] Carpinelli Mazzi M Iavarone A Russo G Musella C Milan G D'Anna F. Mini-Mental state examination: new normative values on subjects in Southern Italy. Aging Clin Exp Res. (2020) 32:699–702. 10.1007/s40520-019-01250-2 31230268

[42] O'Caoimh R Molloy D Fitzgerald C Van Velsen L Cabrita M Nassabi MH. Healthcare recommendations from the personalised ICT supported service for independent living and active ageing (PERSSILAA) study. In: Proceedings of the 3rd International Conference on Information and Communication Technologies for Ageing Well and e-Health, Vol. 1. ICT4AWE (2017). p. 91–103.

[43] O'Caoimh R Molloy DW Fitzgerald C Van Velsen L Cabrita M Nassabi MH. ICT-supported interventions targeting pre-frailty: healthcare recommendations from the personalised ICT supported service for independent living and active ageing (PERSSILAA) study. In: Röcker C, O'Donoghue J, Ziefle M, Maciaszek L, Molloy W, editors. Information and Communication Technologies for Ageing Well and E-Health (ICT4AWE). Porto (2018). 10.5220/0006331800910103

[44] Brouns B Bodegom-Vos LV Kloet AJ Tamminga SJ Volker G Berger MAM. Effect of a comprehensive eRehabilitation intervention alongside conventional stroke rehabilitation on disability and health-related quality of life: a pre-post comparison. J Rehabil Med. (2020) 53:jrm00161. 10.2340/16501977-2785 33369683 PMC8814840

[45] Liguori G American College of Sports Medicine. ACSM's Guidelines for Exercise Testing and Prescription. American College of Sports Medicine (Kingston, RI) (2021).

[46] Liotta G Ussai S Illario M O'Caoimh R Cano A Holland C. Frailty as the future core business of public health: report of the activities of the A3 Action group of the European innovation partnership on active and healthy ageing (EIP on AHA). Int J Environ Res Public Health. (2018) 15:2843. 10.3390/ijerph15122843 30551599 PMC6313423

[47] European Commission. The Silver Economy – Executive Summary. Luxemburg: Publications Office of the European Union (2018).

[48] WE4AHA Project Consortium. Innovation to Market Plan. WE4AHA Project Consortium (2019). Available online at: https://ec.europa.eu/eip/ageing/library/innovation-market-plan_en (accessed January 22, 2021).

[49] Lindner S Illing K Sommer J Krajnc-Nikoli'c T Harer J Kurre C. Development of a binational framework for active and healthy ageing (AHA) bridging austria and slovenia in a thermal spa region. Int J Environ Res Public Health. (2021) 18:639. 10.3390/ijerph18020639 33451090 PMC7828483

[50] Bousquet J Illario M Farrell J Batey N Carriazo AM Malva J. The reference site collaborative network of the European innovation partnership on active and healthy ageing. Transl Med UniSa. (2019) 19:66–81. 31360670 PMC6581486

[51] Programma Mattone Internazionale Salute. EIP-AHA Italy: The Italian experience in the framework of the European Innovation Partnership on Active and Healthy Ageing. Programma Mattone Internazionale Salute (2019). Available online at: https://www.promisalute.it/upload/mattone/gestionedocumentale/Eipaha_Italy_vers_10092019_EN_784_5274.pdf (accessed February 23, 2021).

[52] Lv CM Zhang L. How can collectiveleadership influence the implementation of change in healthcare? Chin Nurs Res. (2017) 4:182e185. 10.1016/j.cnre.2017.10.005

[53] Eckert R West M Altman D Steward K Pasmore B. Delivering a Collective Leadership Strategy for Health Care. London: The King's Fund, Center for Creative Leadership. (2014). Available online at: http://www.kingsfund.org.uk/sites/files/kf/media/delivering-collective-leadership-ccl-may.pdf (accessed February 23, 2021).

[54] Conferenza Stato-Regioni. Accordo ai Sensi Dell'art.4, Comma 1, Del Decreto Legislativo 28 Agosto 1997, n.281, tra il GOVERNO, le Regioni e Le Province autonome di Trento e Bolzano sul Documento Recante “Indicazioni Nazionali per L'erogazione di Prestazioni in Telemedicina.” Rome: Conferenza Stato-Regioni (2020).

[55] McGuinn J Gancheva M Castro R Jones M O'Brien S Markowska A. ESI Funds for Health, Investing for a Healthy and Inclusive EU - Final Report. Luxembourg: Publications Office of the European Union (2019).

[56] Tziraki-Segal C Grimes C Ventura F O'Caoimh R Santana S Zavagli V. Rethinking palliative care in a public health context: addressing the needs of persons with non-communicable chronic diseases. Prim Health Care Res Dev. (2020) 21:E32. 10.1017/S1463423620000328 32928334 PMC7503185

[57] García-Esquinas E Ortolá R Prina M Stefler D Rodríguez-Artalejo F Pastor-Barriuso R. Trajectories of accumulation of health deficits in older adults: are there variations according to health domains? J Am Med Dir Assoc. (2019) 20:710–7.e6. 10.1016/j.jamda.2018.12.023 30772171

[58] Hossin MZ Björk J Koupil I. Early-life social and health determinants of adult socioeconomic position: associations and trends across generations. J Epidemiol Community Health. (2020) 74:412–20. 10.1136/jech-2019-213209 31988239 PMC7307663

[59] Tziraki-Segal C De Luca V Santana S Romano R Tramontano G Scattola P. Creating a culture of health in planning and implementing innovative strategies addressing non-communicable chronic diseases. Front Sociol. (2019) 4:9. 10.3389/fsoc.2019.00009 33869336 PMC8022497

[60] Wenger E McDermott R Snyder W. Cultivating Communities of Practice: A Guide to Managing Knowledge. Boston MA: Harvard Business School Press (2002).

