# Supplementary figures and images for: “One Health” Approach for Health Innovation and Active Aging in Campania (Italy)
Source: Front Public Health. 2021 May 11;9:658959. doi: 10.3389/fpubh.2021.658959 (PMC8144456; doi:10.3389/fpubh.2021.658959)

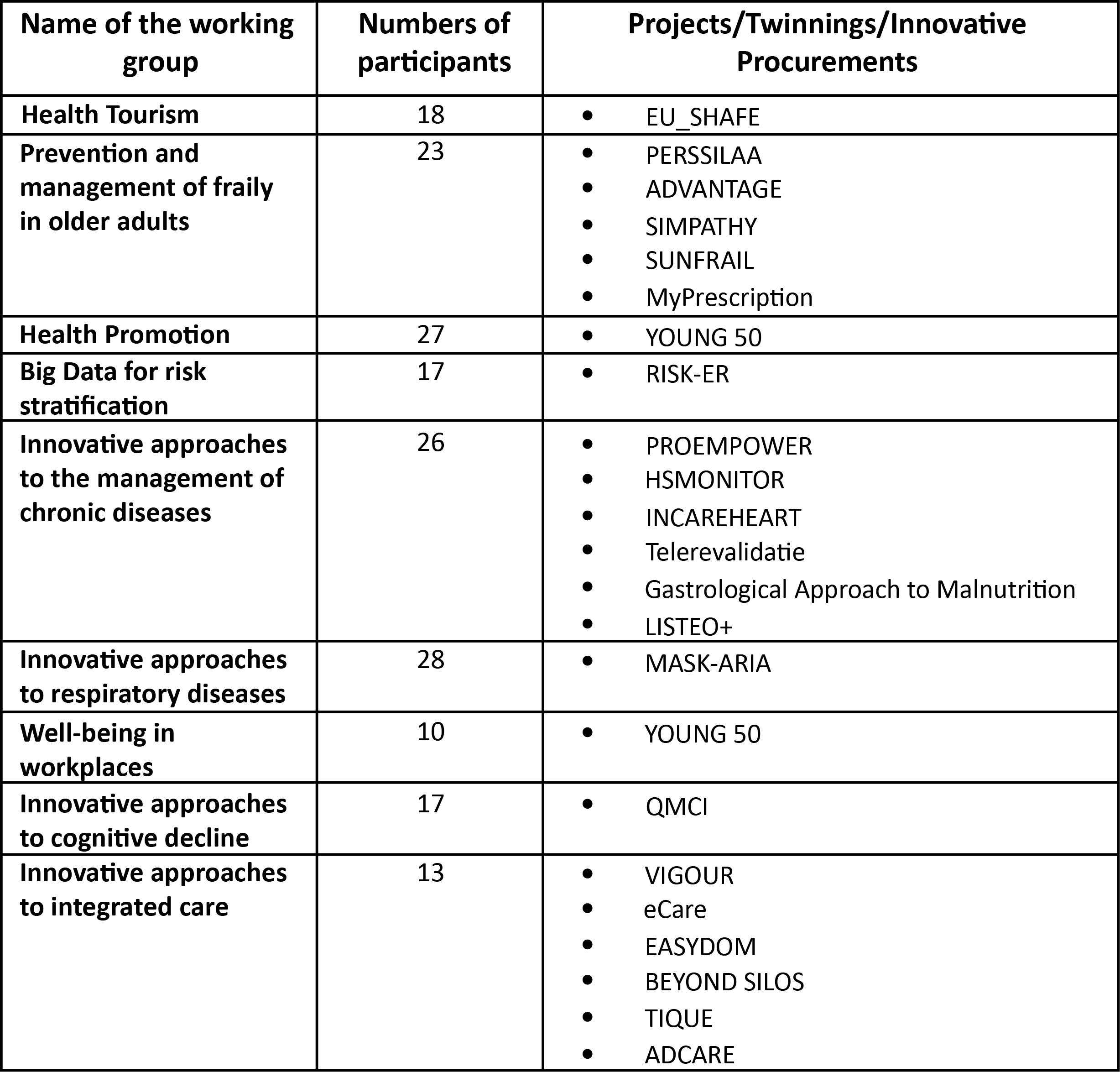

Supplement: Supplementary file 1 [file Image_1.JPEG]

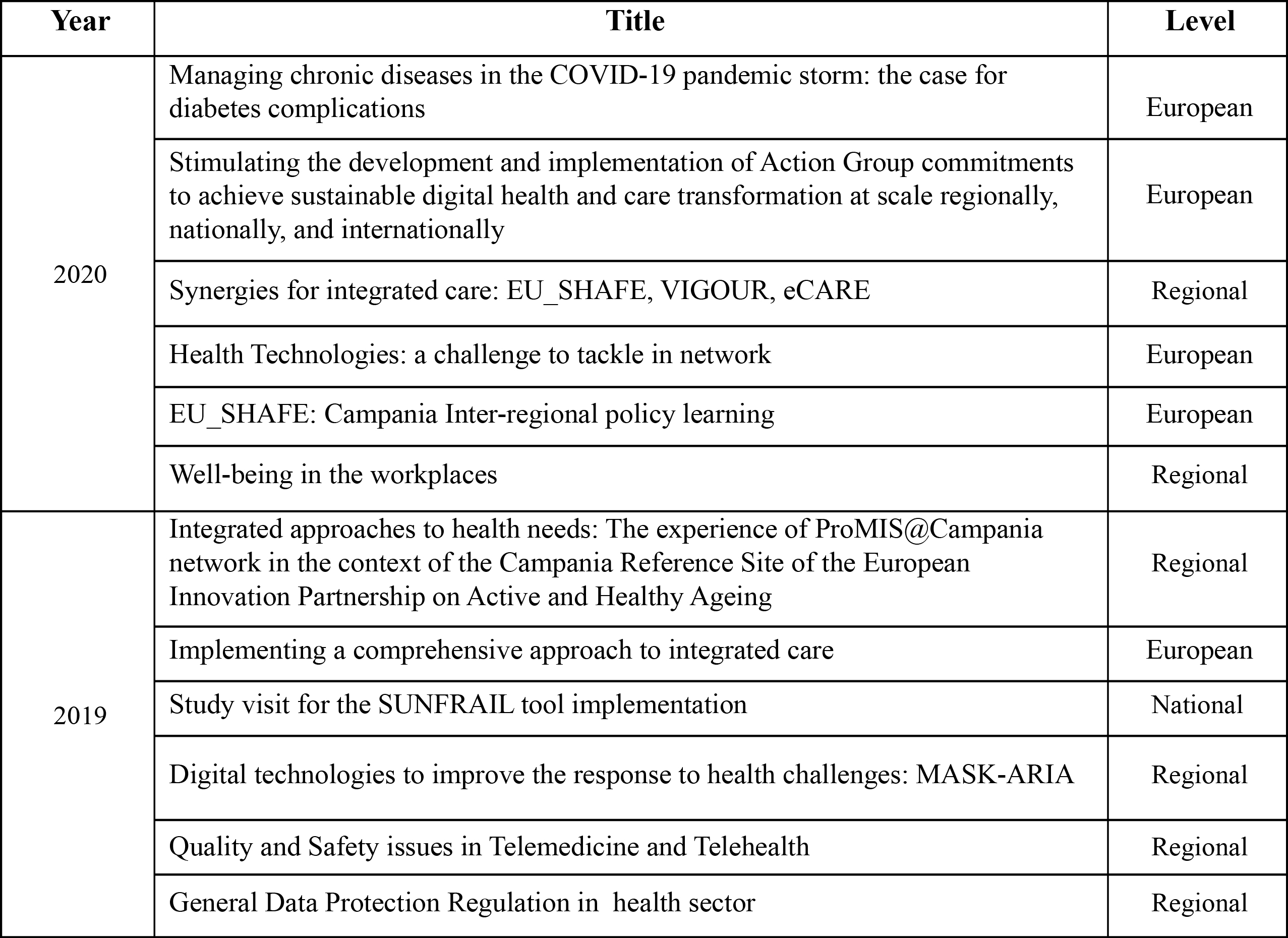

Supplement: Supplementary file 2 [file Image_2.JPEG]
